# Supplementary material for: Long-term outcomes after revascularization in chronic total and non-total occluded coronary arteries: A regionwide cohort study
Source: PLoS One. 2024 Jul 15;19(7):e0307264. doi: 10.1371/journal.pone.0307264 (PMC11249224; doi:10.1371/journal.pone.0307264)
Supplement: S1 Table — (DOCX) [file pone.0307264.s001.docx]

Table S1: Individual components of Charlson comorbidity index

|  | Non CTO | CTO Successful | Unsuccessful |
| --- | --- | --- | --- |
|  | 9065 | 1300 | 273 |
| Charlson Comorbidity index | 3.9 (2.3) | 3.9 (2.3) | 4.4 (2.4) |
| Age categorized |  |  |  |
| < 50 | 577 (6.4%) | 97 (7.5%) | 22 (8.1%) |
| 50-59 | 1574 (17.4%) | 244 (18.8%) | 43 (15.8%) |
| 60-69 | 2810 (31.0%) | 443 (34.1%) | 81 (29.7%) |
| 70-79 | 2900 (32.0%) | 389 (29.9%) | 80 (29.3%) |
| >=80 | 1204 (13.3%) | 127 (9.8%) | 47 (17.2%) |
| Myocardial infarction | 2557 (28.2%) | 392 (30.2%) | 87 (31.9%) |
| Congestive heartfailure | 1476 (16.3%) | 290 (22.3%) | 71 (26.0%) |
| Peripheral vascular disease | 1256 (13.9%) | 161 (12.4%) | 49 (17.9%) |
| Cerebrovascular disease | 1201 (13.2%) | 150 (11.5%) | 39 (14.3%) |
| Dementia | * | * | * |
| Chronic pulmonary disease | 1194 (13.2%) | 143 (11.0%) | 35 (12.8%) |
| Connective tissue disease | 471 (5.2%) | 62 (4.8%) | 10 (3.7%) |
| Ulcer disease | 426 (4.7%) | 48 (3.7%) | 16 (5.9%) |
| Liver disease |  |  |  |
| No liver disease | 8948 (98.7%) | 1287 (99.0%) | 270 (98.9%) |
| Mild | 94 (1.0%) | 10 (0.8%) | 3 (1.1%) |
| Moderate or severe** | 23 (0.3%) | 3 (0.2%) | 0 (0.0%) |
| Diabetes |  |  |  |
| No DM | 7370 (81.3%) | 1042 (80.2%) | 210 (76.9%) |
| Uncomplicated DM | 834 (9.2%) | 129 (9.9%) | 32 (11.7%) |
| Complicated DM | 861 (9.5%) | 129 (9.9%) | 31 (11.4%) |
| Moderate to severe reneal disease |  |  |  |
| No | 8503 (93.8%) | 1220 (93.8%) | 247 (90.5%) |
| Yes | 562 (6.2%) | 80 (6.2%) | 26 (9.5%) |
| Hemiplegia |  |  |  |
| No | * | * | * |
| Yes | * | * | * |
| Cancer kategoriseret til CCI |  |  |  |
| No tumor | 8002 (88.3%) | 1152 (88.6%) | 240 (87.9%) |
| Solid tumor | 951 (10.5%) | 135 (10.4%) | 26 (9.5%) |
| Metastasis | 112 (1.2%) | 13 (1.0%) | 7 (2.6%) |
| Leukemia |  |  |  |
| No | 9023 (99.5%) | 1296 (99.7%) | 273 (100.0%) |
| Yes | 42 (0.5%) | 4 (0.3%) | 0 (0.0%) |
| Lymphoma |  |  |  |
| No | * | * | * |
| Yes | * | * | * |
| AIDS |  |  |  |
| No | * | * | * |
| Yes | * | * | * |
| * Annonymized. ** Grouped | | | |
