# Supplementary material for: Long-term outcomes after revascularization in chronic total and non-total occluded coronary arteries: A regionwide cohort study
Source: PLoS One. 2024 Jul 15;19(7):e0307264. doi: 10.1371/journal.pone.0307264 (PMC11249224; doi:10.1371/journal.pone.0307264)
Supplement: S2 Table — (DOCX) [file pone.0307264.s002.docx]

Table S2: JCTO and Approach

|  | CTO Successful | Unsuccessful |
| --- | --- | --- |
|  | 1300 | 273 |
| JCTO, mean ± SD | 2.48 ± 1.37 | 2.83 ± 1.22 |
| JCTO Redo | 21.7% | 27.5% |
| JCTO in-segment bend >45 | 31.1% | 34.2% |
| JCTO Calcification | 77.3% | 82.9% |
| JCTO Blunt Cap | 53.2% | 64.9% |
| JCTO Lenght > 20 mm | 64.7% | 73.9% |
| JCTO Missing | 611 (47.0%) | 162 (68.4%) |
|  |  |  |
| Antegrade wiring | 70.6% |  |
| Retrograde wiring | 4.1% |  |
| ADR | 11.6% |  |
| RDR | 13.7 |  |
| Approach missing | 686 (52.8%) |  |
