# Supplementary material for: Long-term outcomes after revascularization in chronic total and non-total occluded coronary arteries: A regionwide cohort study
Source: PLoS One. 2024 Jul 15;19(7):e0307264. doi: 10.1371/journal.pone.0307264 (PMC11249224; doi:10.1371/journal.pone.0307264)
Supplement: S3 Table — (DOCX) [file pone.0307264.s003.docx]

Table S3: In-hospital MACCE

|  | Non-CTO | Successful | Unsuccessful |
| --- | --- | --- | --- |
| Any MACE | 254 (2.8%) | 25 (1.92%) | 11 (4.10%) |
| Death | 64 (0.71%) | 1 (0.08%) | 1 (0.37%) |
| Myocardial infarction | 114 (1.26%) | 14 (1.08%) | 5 (1.87%) |
| Stroke | 3 (0.03%) | 0 (0.00%) | 1 (0.37%) |
| Heart failure | 12 (0.13%) | 3 (0.23%) | 2 (0.75%) |
| Repeat revascularization | 70 (0.77%) | 9 (0.69%) | 2 (0.75%) |
