# Supplementary material for: Long-term outcomes after revascularization in chronic total and non-total occluded coronary arteries: A regionwide cohort study
Source: PLoS One. 2024 Jul 15;19(7):e0307264. doi: 10.1371/journal.pone.0307264 (PMC11249224; doi:10.1371/journal.pone.0307264)
Supplement: S4 Table — (DOCX) [file pone.0307264.s004.docx]

Table S4: Variables included in multivariate analysis.

|  | Unadjusted | | |  | Adjusted | | |  |
| --- | --- | --- | --- | --- | --- | --- | --- | --- |
|  | without imputation | P | with imputation | p | without imputation | p | with imputation | p |
| CTO Successful | 1.03 (0.94 ;1.12) | 0.556 | 1.03 (0.94 ;1.12) | 0.556 | 0.94 (0.85;1.05) | 0.259 | 0.98 (0.90;1.07) | 0.710 |
| CTO Unsuccessful | 1.42 (1.21 ;1.67) | <0.001 | 1.42 (1.21 ;1.67) | <0.001 | 1.21 (0.99;1.48) | 0.069 | 1.22 (1.04;1.43) | 0.002 |
| Age | 1.04 (1.03;1.04) | <0.001 | 1.04 (1.03;1.04) | <0.001 | 1.02 (1.02;1.02) | <0.001 | 1.02 (1.02;1.02) | <0.001 |
| BMI 18-25 | 0.54 (0.43;0.67) | <0.001 | 0.56 (0.45;0.71) | <0.001 | 0.54 (0.42;0.69) | <0.001 | 0.62 (0.50;0.78) | <0.001 |
| BMI > 25 | 0.47 (0.38;0.59) | <0.001 | 0.49 (0.39;0.62) | <0.001 | 0.49 (0.38;0.63) | <0.001 | 0.58 (0.47;0.73) | <0.001 |
| Previous Revascularization | 1.43 (1.34;1.53) | <0.001 | 1.45 (1.36;1.54) | <0.001 | 1.24 (1.14;1.33) | <0.001 | 1.20 (1.12;1.28) | <0.001 |
| Previous smoker | 1.21 (1.12;1.30) | <0.001 | 1.21 (1.12;1.30) | <0.001 | 1.14 (1.05;1.24) | 0.003 | 1.12 (1.04;1.21) | <0.001 |
| Active smoker | 1.23 (1.13;1.34) | <0.001 | 1.25 (1.15;1.36) | <0.001 | 1.42 (1.29;1.58) | <0.001 | 1.36 (1.25;1.49) | <0.001 |
| Statin treatment | 0.84 (0.79;0.91) | <0.001 | 0.84 (0.79;0.91) | <0.001 | 0.81 (0.74;0.89) | <0.001 | 0.80 (0.74;0.86) | <0.001 |
| CKD G2 | 1.09 (0.99;1.19) | 0.094 | 1.06 (0.97;1.18) | 0.167 | 0.83 (0.74;0.93) | 0.002 | 0.82 (0.74;0.91) | <0.001 |
| CKD G3a | 1.27 (1.15;1.41) | <0.001 | 1.25 (1.13;1.38) | <0.001 | 0.82 (0.72;0.94) | 0.004 | 0.81 (0.72;0.91) | <0.001 |
| CKD G3b | 2.12 (1.89;2.37) | <0.001 | 2.03 (1.81;2.37) | <0.001 | 1.02 (0.88;1.19) | 0.780 | 0.99 (0.87;1.13) | 0.880 |
| CKD G4 | 3.67 (3.18;4.24) | <0.001 | 3.13 (2.71;3.61) | <0.001 | 1.39 (1.14;1.69) | 0.001 | 1.36 (1.16;1.60) | 0.001 |
| CKD G5 | 3.92 (3.23;4.75) | <0.001 | 3.72 (3.06;4.52) | <0.001 | 1.77 (1.39;2.25) | <0.001 | 1.61 (1.30;1.99) | <0.001 |
| 2-vessel disease | 1.42 (1.33;1.52) | <0.001 | 1.42 (1.33;1.52) | <0.001 | 1.25 (1.15;1.36) | <0.001 | 1.23 (1.15;1.32) | <0.001 |
| 3-vessel disease | 1.97 (1.83;2.11) | <0.001 | 1.97 (1.83;2.11) | <0.001 | 1.39 (1.27;1.52) | <0.001 | 1.42 (1.32;1.54) | <0.001 |
| EF 40-50 | 0.77 (0.68;0.87) | <0.001 | 0.75 (0.67;0.85) | <0.001 | 0.81 (0.70;0.93) | 0.003 | 0.83 (0.73;0.93) | <0.001 |
| EF > 50 | 0.43 (0.39;0.47) | <0.001 | 0.44 (0.40;0.47) | <0.001 | 0.64 (0.58;0.70) | <0.001 | 0.65 (0.60;0.70) | <0.001 |
| NIDM | 1.32 (1.21;1.44) | <0.001 | 1.32 (1.21;1.44) | <0.001 | 1.04 (0.93;1.16) | 0.470 | 1.07 (0.98;1.17) | 0.120 |
| IDM | 1.71 (1.56;1.87) | <0.001 | 1.71 (1.56;1.87) | <0.001 | 1.29 (1.15;1.47) | <0.001 | 1.24 (1.12;1.38) | <0.001 |
| Charlson comorbidity index | 1.24 (1.23;1.25) | <0.001 | 1.24 (1.23;1.25) | <0.001 | 1.13 (1.11;1.15) | <0.001 | 1.13 (1.11;1.15) | <0.001 |
| Previous MI | 1.47 (1.39;1.56) | <0.001 | 1.47 (1.39;1.56) | <0.001 |  |  |  |  |
| IHD in family | 0.82 (0.77;0.87) | <0.001 | 0.82 (0.77;0.87) | <0.001 |  |  |  |  |
| Sex | 0.98 (0.92;1.05) | 0.569 | 0.98 (0.92;1.05) | 0.569 |  |  |  |  |
