# Supplementary material for: Long-term outcomes after revascularization in chronic total and non-total occluded coronary arteries: A regionwide cohort study
Source: PLoS One. 2024 Jul 15;19(7):e0307264. doi: 10.1371/journal.pone.0307264 (PMC11249224; doi:10.1371/journal.pone.0307264)
Supplement: S5 Table — (DOCX) [file pone.0307264.s005.docx]

Table S5: Sensitivity analysis

|  | Non-CTO vs. Successful CTO | | | Non-CTO vs. Unsuccessful CTO | | |
| --- | --- | --- | --- | --- | --- | --- |
|  | Unadjusted HR | Adjusted HR | P | Unadjusted HR | Adjusted HR | p |
| MACCE | 1.03 (0.94 ;1.12) | 0.98 (0.90;1.07) | 0.71 | 1.42 (1.21 ;1.67) | 1.22 (1.04;1.43) | <0.01 |
| MACCE Complete case |  | 0.94 (0.85;1.04) | 0.26 |  | 1.21 (0.99;1.48) | 0.07 |
| MACCE without staged patients | 1.03 (0.94;1.14) | 0.97 (0.88;1.07) | 0.56 | 1.43 (1.20;1.69) | 1.20 (1.01;1.42) | 0.04 |
| MACCE interaction sex and age | 1.08 (0.99;1.18) | 0.98 (0.90;1.08) | 0.73 | 1.43 (1.22;1.69) | 1.20 (1.02;1.42) | 0.03 |
| MACCE only complete revasc | 1.20 (1.10;1.31) | 1.07 (0.97;1.18) | 0.17 | 1.65 (1.40;1.95) | 1.32 (1.11;1.57) | <0.001 |
| MACCE without unsuccessful non-CTO | 1.05 (0.96;1.15) | 1.00 (0.92;1.09) | 0.95 | 1.45 (1.24;1.70) | 1.24 (1.06;1.46) | <0.001 |
| Adjusted for calendar year | 1.03 (0.94;1.12) | 0.99 (0.90;1.08) | 0.81 | 1.41 (1.20;1.66) | 1.20 (1.01;1.41) | 0.04 |
| MACCE propensity score adjustment |  | 0.93 (0.86;1.02) | 0.14 |  | 1.21 (1.02;1.42) | 0.03 |
|  |  |  |  |  |  |  |
| MACCE without revascularizations |  |  |  |  |  |  |
| 0 to 9 years | 1.04 (0.94;1.14) | 1.02 (0.93;1.12) | 0.66 | 1.55 (1.30;1.83) | 1.34 (1.13;1.59) | <0.001 |
| 0 to 30 days | 0.84 (0.62;1.14) | 0.79 (0.58;1.08) | 0.13 | 2.20 (1.56;3.32) | 1.67 (1.10;2.54) | 0.02 |
| 30 to 365 days | 0.89 (0.70;1.14) | 0.78 (0.61;1.01) | 0.06 | 1.62 (1.08;2.42) | 1.21 (0.81;1.83) | 0.33 |
| 1 to 9 years | 1.10 (0.99;1.23) | 1.11 (1.00;1.24) | 0.05 | 1.42 (1.15;1.75) | 1.30 (1.05;1.61) | 0.02 |
