# Supplementary material for: Long-term outcomes after revascularization in chronic total and non-total occluded coronary arteries: A regionwide cohort study
Source: PLoS One. 2024 Jul 15;19(7):e0307264. doi: 10.1371/journal.pone.0307264 (PMC11249224; doi:10.1371/journal.pone.0307264)
Supplement: S6 Table — (DOCX) [file pone.0307264.s006.docx]

Table S6: Definition of other indication

| Indication | Non-CTO | Success | Unsuccess |
| --- | --- | --- | --- |
| Arrhythmia | 298 (3.3%) | 35 (2.7%) | 25 (9.2%) |
| PCI before valve surgery | 88 (1%) | 5 (0.4%) | 3 (1.1%) |
| Complication to CAG, PCI or CABG | 23 (0.3%) | 0 (0.05) | 0 (0.0%) |
| Non-anginal chest pain | 26 (0.3%) | 3 (2.3%) | 3 (1.1%) |
| PCI before non-cardiac surgery | 17 (0.2%) | 1 (0.1%) | 1 (0.4%) |
| Hybrid procedure after CABG | 91 (1.0%) | 3 (0.2%) | 3 (1.1%) |
| Missing | 108 (1.2%) | 15 (1.2%) | 7 (2.6%) |
