# Supplementary figures and images for: Long-term outcomes after revascularization in chronic total and non-total occluded coronary arteries: A regionwide cohort study
Source: PLoS One. 2024 Jul 15;19(7):e0307264. doi: 10.1371/journal.pone.0307264 (PMC11249224; doi:10.1371/journal.pone.0307264)

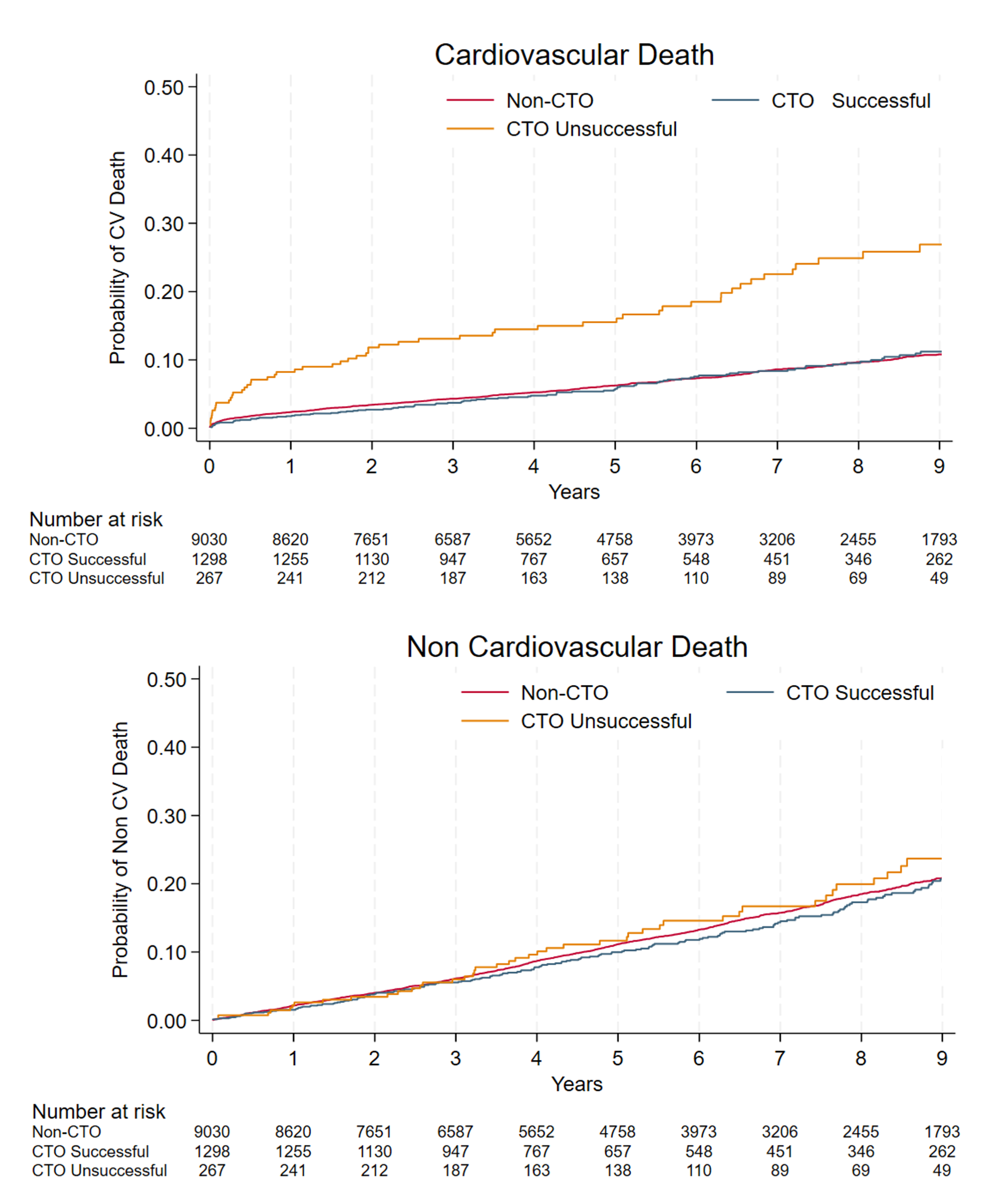

Supplement: S1 Fig — (TIF) [file pone.0307264.s007.tif]

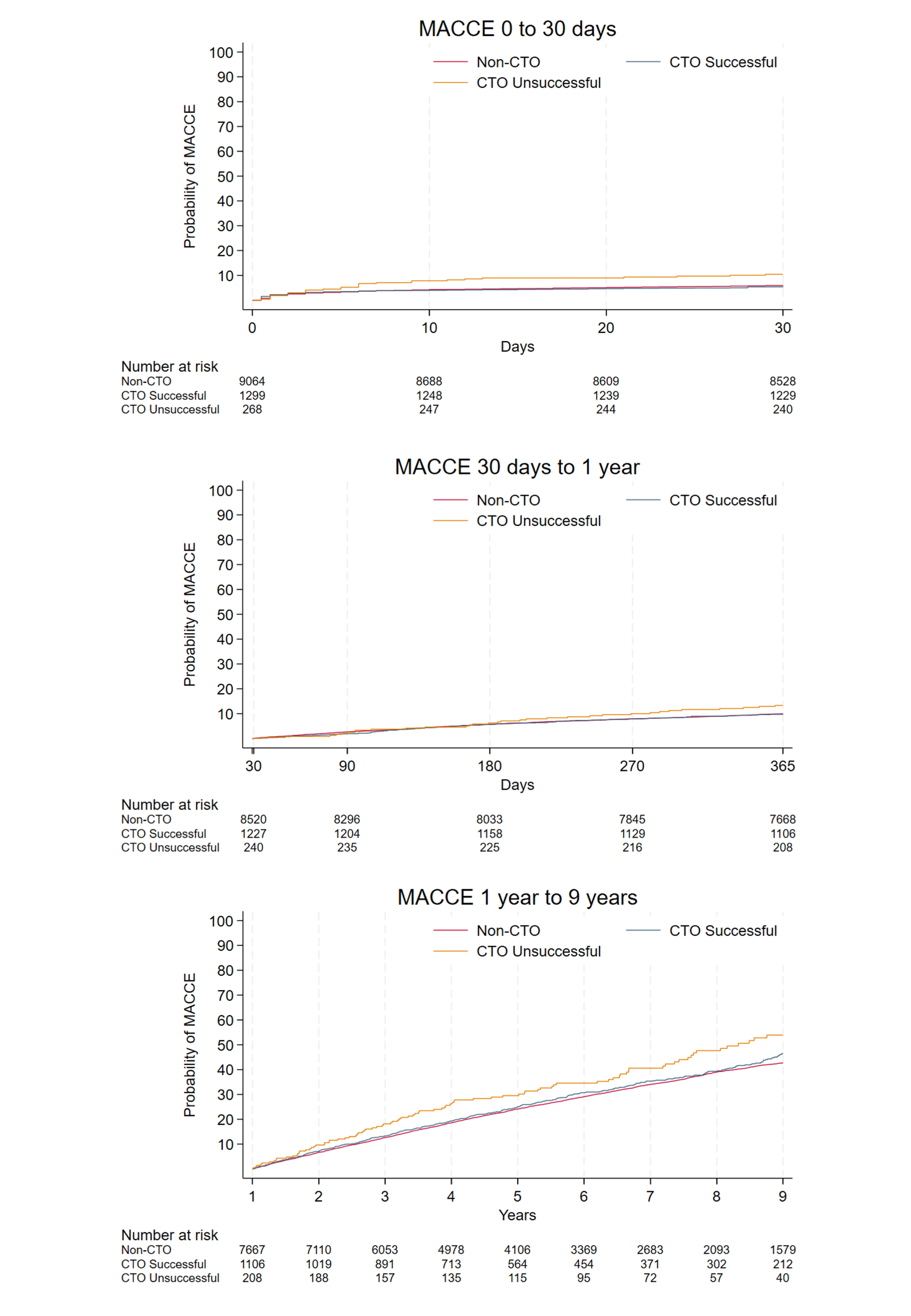

Supplement: S2 Fig — (TIF) [file pone.0307264.s008.tif]

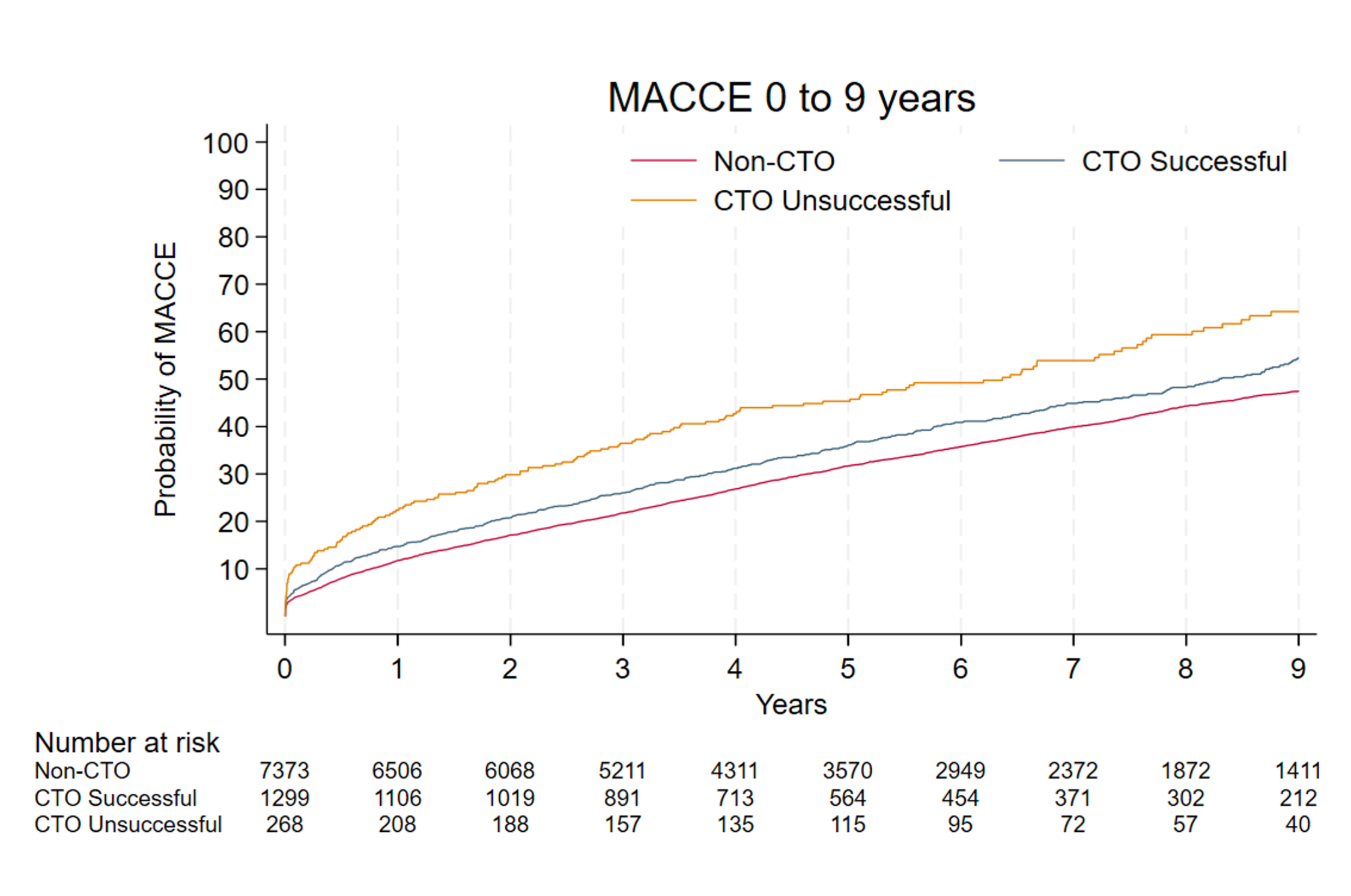

Supplement: S3 Fig — (TIF) [file pone.0307264.s009.tif]
