# Supplementary material for: Long-term outcomes after revascularization in chronic total and non-total occluded coronary arteries: A regionwide cohort study
Source: PLoS One. 2024 Jul 15;19(7):e0307264. doi: 10.1371/journal.pone.0307264 (PMC11249224; doi:10.1371/journal.pone.0307264)
Supplement: Attachment — Submitted filename: Response to Reviewers.docx [file pone.0307264.s010.docx]

**Review Comments to the Author**

**Reviewer #1: This manuscript is a large-sample, long-term follow-up observational study. The findings seem to be a little bit interesting, but the statistical methods could be further refined. I think the results would have been more convincing if the authors could have done the propensity score matching analysis, as the number of patients in the three groups currently varies considerably (non-CTO 9065 patients; successful CTO 1300 patients and non-successful CTO 273 patients).**

First, we thank the reviewer for these statistical considerations. Before embarking of the study, we had extensive considerations on which statistical methods to address which is also on of the reasons for that a statistician is co-authoring the paper (LJHM). As the reviewer points out propensity score matching (PSM) was an option. However, it was not chosen because it is our belief that it will induce more bias than conventional co-variate adjustment. This is elaborated below.

1) PSM is often used in the situation where not all data in a population is present. However, in this analysis we have access to the entire population who were treated with PCI in the Central Denmark Region with complete follow-up through nationwide registries.
2) The problem with using PSM in this setting, as in many other registries, is that we will lose power because some patients will be unmatched due to missing values on co-variates. In this study the percentage was 25.4% in the successful group, and 35.6% in the unsuccessful group. This would lead to a lower power and therefore more imprecise estimates.
3) The issue with missing values were in this study solved with multiple imputation assuming that data were missing at random (MAR). As it have previously been shown this may influence the bias in PSM, and again lead to a biased estimate (Choi et al, Eur J Epidemiol 2019, DOI: [10.1007/s10654-018-0447-z](https://doi.org/10.1007%2Fs10654-018-0447-z))
4) Another problem with PSM is that it would be necessary to perform a very sophisticated analysis with three groups which would probably lead to many patients not being matched unless a high caliper was used. Usage of a high caliper is associated to an increased risk of residual confounding. Since there is a large scatter of propensity scores between the three groups, this will most likely induce a higher degree of confounding and therefore have the opposite effect than the anticipated (Lunt, Am J Epidemiol 2014, DOI: 10.1093/aje/kwt212).
5) Also, as pointed out by Elze and colleageus in JACC 2017 ( DOI: <https://doi.org/10.1016/j.jacc.2016.10.060>) most of the propensity score models only vary <5% as compared to complete co-variate analysis and therefore it is recommended to use complete covariate adjustment when there is sufficient power. Power is not an issue in this study as there is more than 4,000 events and 155 in the unsuccessful group.
6) Finally, we are not aware of any studies supporting that because there is a variation of n between the group the usage of PSM would lower bias unless it is due to insufficient power.

PSM is performed from a propensity score, and we do acknowledge the usage of propensity scores for adjusting in a survival analysis. Therefore, we made a sensitivity analysis with adjustment of a propensity score calculated from the co-variates instead of the full co-variate list. The potential advantage would be a more precise estimate due to a lower standard error, yet this was not the case in our analysis due to a sufficient power for conventional co-variate adjustment. The point estimate was 5.1% lower in the successful group and 0.8% lower in the unsuccessful group in the multiple imputation analysis. In the complete case analysis, the estimate was 3.2% lower in the successful group and 2.5% lower in the unsuccessful group. The significance level did not change in any of the analysis. It was decided to use the conventional analysis as the main analysis for three reasons. Firstly, propensity score analysis is more prone to bias if data are missing at random instead of missing completely at random. Secondly, interpretation of covariates is not feasible in a propensity score analysis. Thirdly, the study did no lack power.

All the above is verified by a statistician (LJHM).

Added line 198: Adjusting for a propensity score calculated from the full co-variate list resulted in a HR 95%CI in the successful group of 0.93 (0.86;1.02) and 1.21 (1.02;1.42) in the unsuccessful group

**Reviewer #2:**

**1) It would be good to deliver data concerning SYNTAX Score II, Euroscore, and eventually STS**

We agree that especially the SYNTAX score would better characterize the coronary anatomy in the study. Unfortunately, these data were not available. Standard practice for all patients in the study was heart team conference if anatomy was suited for CABG. The Euroscore and STS score are validated for post cardiac surgery mortality and therefore the data does not provide prognostic implications for our patient group. Both the Euroscore 2 and STS are surrogates for comorbidities and in our study, we used the charlson comorbidity index to assess the meaning of comorbidities for outcome. It has been added to limitations that SYNTAX score is missing.

Added line 303: “Secondly, SYNTAX score was not recorded in WDHR.”

**2) What about investments procedures, did authors include patients after last procedure (if it was successfull or not), sometimes it takes even 3 or 4 times before final decision (procedural failure or success)**

Thank you for this valuable comment. In table 2 it is stated that 12.3% and 9.5% of patients in the successful and unsuccessful group underwent a staged procedure and that the number of procedures was 1.2 ± 0.3 and 1.1 ± 0.3 in these groups. Staged procedure was defined as patients undergoing one or more procedures within ninety days of the previous CTO procedure (line 96).

For clarity, “investment” has been added as a synonym to “staged” in the text.

Added line 96: (investment)

**3) Table 2 - lesion characteristics - what deas it mean that tere was 1-, 2- and 3- vesse disease? Does it mean that cto was that one which was treated at the end, or there were two CTOs?**

Thank you for the ability to clarify. It indicates the number of native coronary vessels that have been treated with revascularization or had a diameter stenosis > 50%. A note to table 2 has been added for clarity.

**4) Neo with meta in unsuccessful group was 2.6 x higher than in the CTO successful group? similar situation was with heart failure? Do the authors think that this could have impacted on MACCE and mortality difference between both groups, which was greater in the unsuccessful group???**

This is a very relevant question. We did adjust for CCI which incorporated neoplasms, and LVEF in the adjusted analysis to account for these as a possible confounder (supplementary table 4). As observed in the adjusted analysis both CCI and LVEF was significantly associated to a higher risk.

**Reviewer #3: In this paper, the authors assessed the outcomes of patients undergoing successful and unsuccessful CTO PCI compared to patients with non-CTO PCI. They found that primary outcome of MACCE was higher in unsuccessful CTO PCI group compared to non-CTO PCI group but similar in the successful CTO PCI group compared to non-CTO PCI group. They also performed sensitivity analysis excluding revascularization, and results were similar.**

**Minior points:**

1. **Please add how the complete revascularization was defined.**

Thanks for the opportunity to clarify this. Completely revascularized is defined as no remnant stenosis >50% in any of the native vessels. This have been clarified in the text.

Added line 205: Restricting the analysis to patients with complete revascularization in all stenosis <50% in the non-CTO group showed an adjusted MACCE HR (95%CI): 1.07 (0.97;1.18) in the successful CTO group and HR (95%CI): 1.32 (1.11;1.57) in the unsuccessful CTO group (supplementary figure 3, supplementary table 5).

**Reviewer #4: In this paper, Holck and colleagues sought to investigate the prognostic impact of unsuccessful CTO PCI in a long term follow-up using a regionwide cohort. We understand that CTO PCI is still a field of debate even after RCT dedicated to this area, once all of RCT published are underpowered for hard endpoints. Thus, good quality registries are important in this gap of knowledge. Some issues regarding this paper are bellow:**

**1) The novelty (or the lack of novelty) of this paper should be highlighted. Some analyses compering successful versus unsuccessful CTO PCI have been published since 20 years ago and we would like to know how this paper add new information in the gaps about CTO PCI.**

As the reviewer point out there is several studies investigating successful versus unsuccessful CTO revascularization which is also mentioned in the discussion section (megaly et al 2022, cardiovasc interv ther). Unfortunately, all these studies have failed to provide an actual control group consisting of patients without CTO lesions. This is important since it is debatable whether CTO patients should be treated as non-CTO CCS patients. One attempt was made by Almarzooq and colleagues, but they only investigated successfully treated CTO patients. Another attempt was made by Azzalini and colleagues who unfortunately only reported a confounder adjusted estimate for the successful group and not for the unsuccessful group. It is our opinion that our study emphasizes and confirm previous findings that successfully revascularized CTO patients have equal risk of MACCE as non-CTO patients. Furthermore, our findiings add that despite effective confounder adjustment unsuccessfully treated CTO patients still exhibit an increased risk of MACCE in comparison with non-CTO patients. We have revised the first paragraph of the discussion to highlight this.

Added line 229: “The findings of our study confirms that successfully revascularized CTO patients have outcome comparable with non-CTO patients. Secondly, we provide a confounder adjusted estimate for patients with unsuccessful CTO treatment in comparison with non-CTO patients as an addon to previous investigation. This supports the findings of previous studies on successful versus unsuccessful CTO PCI which failed to include a non-CTO control group [12]. Thirdly, the long-term results emphasize that the estimate in previous studies may be exaggerated due to short follow-up time.”

**2) Nature of this kind of analysis lead to 3 very different populations. I understand that a propensity score matching analysis would be more appropriate than just an adjusted analysis. Could the authors comment?**

Thanks for the question. Please see response to reviewer 1.

**3) Range of inclusions of patients is wide (2009-2019) and some improvements in PCI technology (2nd generation DES and duration of DAPT, for example) were acquired in this period. Any type os statistical test was performed to investigate the impact of inclusion time (past versus recent) in the results? If not, more details about type of stents would be useful.**

This is a very valid point since several CTO algorithms have been introduced in this period, and new equipment and stents have been implemented. The success rate before the implementation of the hybrid algorithm (2015) was 76.2%, and after it was 83.1%. Therefore, we did also perform a sensitivity analysis adjusting for treatment year to see if this would alter the point estimate (supplementary table 5). Very minor changes in the point estimate were observed. Our interpretation was that that the risk of confounding was minor, and it was therefore not pursued further.

|  | Non-CTO vs. Successful CTO | | | Non-CTO vs. Unsuccessful CTO | | | | | |  |
| --- | --- | --- | --- | --- | --- | --- | --- | --- | --- | --- |
|  | Unadjusted HR | Adjusted HR | P | | Unadjusted HR | | Adjusted HR | | p | |
| MACCE | 1.03 (0.94;1.12) | 0.98 (0.90;1.07) | 0.71 | 1.42 (1.21;1.67) | | 1.22 (1.04;1.43) | | <0.01 | |  |
| Adjusted for calendar year |  | 0.99 (0.90;1.08) | 0.81 |  | | 1.20 (1.01;1.41) | | 0.04 | |  |

Added to line 198: Complete case analysis, exclusion of staged patients and adjustment for treatment year only caused minor changes in the point estimate (supplementary table 5).

**4) Definition of MI should be clarified. We know that it has been modified across last 10 years and we do not know if MI in patients enrolled in 2009 is the same as in patients in 2019. It would be useful to understand the lower risk of MI in patients with unsuccessful CTO PCI.**

The definitions used nationwide in Denmark during the study period (2009 to 2021) was the second, third and fourth universal definition. In Denmark the nationwide diagnosis of Type I and II MI have been based upon high sensitivity troponin since 2010 and before that fourth-generation troponin assays. Therefore, we do not believe that this should differ during the follow-up time. MI was adjudicated by the treating physician and the positive predictive value of first-time MI in the Danish national patient registry (which was used in the analysis) is 99% and 88% for recurrent MI (sundboll et al 2016, BMJ open). The obvious limitation is that procedural MI was not assessed routinely and therefore the divison in spontaneous MI and procedural MI was not feasible.

Added line 113: Nationwide analysis of Troponin I or T was used during the study period to diagnose MI.

Added line 303: Procedural MI was not assessed routinely, and it was not possible to include this in MACCE.

**5) I agree with the last sentence in conclusion: These findings suggest that CTO procedures should only be offered in dedicated programs with high success rates. However, given the nature pf this paper, I think that causality is not an answer here. Unsuccessful CTO PCI marks a complex patient, older, with less LVEF, more diabetes and G3 CKD. Thus, I understand that (and this paper do not have power to undo my point) is this myriad of characteristics and not only the unsuccessful procedure the leads to worse prognosis. Finally, high success rates are associated to better prognosis not only because high level techniques, but firstly selecting the more appropriate patient (appropriate indication and technique). The inclusion of CTO patients in OMT (not because unsuccessful PCI) in this analysis could help to understand these remaining questions. But I agree with authors that is not the main question if this paper. I suggest to remove this sentence of the conclusion (at least in abstract) once the eventual reader could misunderstand this speculative point.**

We thank the reviewer for these insightful reflections. We agree that there could be a risk of confounding by indication in the unsuccessful group. This was also why an OMT group was not included since they would be at very high risk of confounding by indication. This was also explored by Park et al., JAHA, 2021 who observed that failed CTO PCI patients had better prognosis than OMT patients. We will follow the reviewer’s recommendation and remove the speculative sentence from the conclusion.

Abstract conclusion changed from:

“The pre-specified hypothesis was accepted. Successful CTO PCI was associated with equivalent long-term outcomes as non-CTO PCI, and unsuccessful CTO PCI was identified as a high-risk group associated to worse outcomes. These findings suggest that CTO procedures should only be offered in dedicated programs with high success rates.”

To

“The pre-specified hypothesis was accepted. Successful CTO PCI was associated with equivalent long-term outcomes as non-CTO PCI, and unsuccessful CTO PCI was identified as a high-risk group associated to worse outcomes.”

Conclusion change from

“Unsuccessfully treated CTO patients is a high-risk group and improving technical success rate and lowering procedural risk may be beneficial. The findings may improve individualization of treatment and suggests a need for dedicated CTO programs.”

To

“The findings may improve individualization of treatment and suggests a need for dedicated CTO programs. However, future research should focus on randomizing CTO patients to overcome the inherent risk of observational data.”

**Reviewer #5: I have reviewed the manuscript entitled “Long-term Outcomes After Revascularization in Chronic Total and Non-Total Occluded Coronary Arteries: A Regionwide Cohort Study”. In this article, the authors enrolled all consecutive patients undergoing PCI in the Central Region of Denmark from 2009 to 2019. They concluded that successful CTO PCI was associated with equivalent long-term outcomes as non-CTO PCI, and unsuccessful CTO PCI was identified as a high-risk group associated to worse outcomes.**

**The overall topic of this study is appealing and sought to provide important insights into the long-term outcomes of CTO-PCI. However, some key issues still need to address and consider:

1. The definition of CTO lesion should be more accurate in detail, not only "believed to have persisted for at least three months".**

The CTO definition complied to the Academic Research Consortium consensus statement: “*the 2 key characteristics of a CTO are an occlusion with absence of antegrade flow through the lesion with a presumed or documented duration of ≥3 months.”* This means that both definite and probable CTO’s were include in the study.

The paragraph in the methods section have been modified to reflect the ARC-CTO consensus more accurate.

Added line 93: without any antegrade flow (TIMI 0) and the absence of thrombus. As according to the definition of the Academic Research Consortium the occlusion had to have a documented or presumed duration ≥ 3 months.

**2. The major comparison of this study is non-successful CTO PCI with successful CTO PCI and non-CTO PCI, but how about those success or non-success non-CTO PCI?**

This is a very interesting research question but not the scope of this paper. In total 1.6% of the non-CTO patients had a failed index procedure. These patients had a very high risk in comparison to the other groups, and by incorporating them as a specific group in the analysis an adjusted HR 95% CI of 2.91 (2.41;3.53) was observed. Therefore, we performed a sensitivity analysis excluding these patients from MACCE. This analysis only did minor changes to the point estimate and did not change the significance level (table below).

|  | Non-CTO vs. Successful CTO | | | Non-CTO vs. Unsuccessful CTO | | | | | |  |
| --- | --- | --- | --- | --- | --- | --- | --- | --- | --- | --- |
|  | Unadjusted HR | Adjusted HR | P | | Unadjusted HR | | Adjusted HR | | p | |
| MACCE | 1.03 (0.94;1.12) | 0.98 (0.90;1.07) | 0.71 | 1.42 (1.21;1.67) | | 1.22 (1.04;1.43) | | <0.01 | |  |
| MACCE without unsuccessful non-CTO | 1.05 (0.96;1.15) | 1.00 (0.92;1.09) | 0.95 | 1.45 (1.24;1.70) | | 1.24 (1.06;1.46) | | <0.01 | |  |

Added line 207: Excluding patients with failed non-CTO PCI did not change the point estimate significantly (supplementary figure 3, supplementary table 5).

**3. Why select MACCE but not MACE as the primary endpoint, as the rate of stroke is not expected to be higher in CTO, or non-CTO cohort.**

Stroke was included in MACCE as it is a potential complication during the CTO PCI procedure. Since it has been observed as a rare but disabling complication. It is also recommended by the ARC-CTO to include as a safety end point in CTO trials (Ybarra, Circulation 2021).

**4. What’s the follow-up rate between three arms, which is very crucial for this long-term analysis, and how to deal with those patients with the missing outcomes should be described clearly in the statistics section.**

Follow-up time was consistent across the three groups (median IQR: non-CTO: 6.0 [3.5;9.1], success CTO: 5.7 [3.6;8.9], unsuccessful CTO: 5.6 [2.9:8.6]). The slightly lower follow-up time in the CTO groups was due to more deaths. Of the 36 (0.3%) patients lost to follow-up, 4 were in the successful CTO group and the rest in the non-CTO group. The median follow-up time in patients lost to follow-up was 3.1 [1.6;5.9] years. Patients contributed risk time until lost-to-follow-up and were censored at lost-to-follow-up which is stated in the statistics section. However, it has been changed for clarity.

Line 137: The primary and secondary endpoints were followed from the date of the index procedure or the last staged procedure until emigration, occurrence of an event or December 1, 2021, which ever came first.

Changed to

The primary and secondary endpoints were followed from the date of the index procedure or the last staged procedure and censored at emigration (loss-to-follow-up), occurrence of an event or December 1, 2021, which ever came first.

Stratified follow-up time have been added to the results section.

Added to line 169: (non-CTO: 6.0 [3.5;9.1], success CTO: 5.7 [3.6;8.9], unsuccessful CTO: 5.6 [2.9:8.6])

**5. The difference was driven by an increased risk of all-cause death and CV death, that hard to explain what the reason of CV death, as the rate of MI and revascularization were lower in non-successful CTO patients.**

This is discussed in the discussion line 268 to 274. Possible explanations supported by the literature may be higher risk of malignant arrhythmias or fatal myocardial infarction.

**6. In the result section, the statement looks underdetermined, as the upper and lower bounds of 95%CI were from <1.0 through >1.0. [Successful CTO PCI was associated to lower event rates within the first year (HR (95% CI) 0-30 days:0.86 (0.67;1.11), 30-365 days: 0.84 (0.69;1.03), 1-9 years: 1.06 (0.95;1.19)), and unsuccessful was only associated to higher events rates at very short term follow-up and long term (HR (95%CI) 0-30 days: 1.39 (0.94;2.05), 30-365 days: 1.04 (0.73;1.49), 1-9 years: 1.23 (0.99;1.52))]**

It is true that the 95% CI incorporate 1 and therefore it is not statistically significant at a significance level of 5%. As this is an explorative analysis of which time-intervals that may influence the primary end point the most the point estimate may be of greater importance than the 95%CI. We have added to the text that this was not statistically significant (line 188 and 190).

**7. What’s the definition of MI, including peri-procedural MI and spontaneous MI, what’s the rate of each component during follow-up?**

The definitions used nationwide in Denmark during the study period (2009 to 2021) was the second, third and fourth universal definition. In Denmark the nationwide diagnosis of Type I and II MI have been based upon high sensitivity troponin since 2010 and before that fourth-generation troponin assays. MI was adjudicated by the treating physician and the positive predictive value of first-time MI in the Danish national patient registry (which was used in the analysis) is 99% and 88% for recurrent MI (sundboll et al 2016, BMJ open). Peri-procedural biomarkers are not routinely collected in Denmark and therefore was peri-procedural MI not assessed in the study. Also, to be certain the PMI is associated to death the SCAI-definition should be used, and CK-MB is very rarely used in Denmark after implementation of Troponin I and T. As a proxy in-hospital MI was assessed and did no differ between the groups (Non-CTO: 1.26%, successful CTO: 1.08% and unsuccessful 1.87%). This have been added to limitations.

Added line 303: Secondly, SYNTAX score was not recorded in WDHR. Procedural MI was not assessed routinely, and it was not possible to include this in MACCE.

**8. The unit of radiation in table 2?**

This have been added.

**9. Theoretically, all the CTO cases were CCS, how about those UAP patients in table 2, and what’s the definition of “other” indication？**

The main reason for other was PCI due to an Arrhythmia. Other reasons were PCI before valve surgery, complication to other cardiac procedure, non-anginal chest pain, non-cardiac surgery, hybrid procedure, and in a minority of cases it was missing. A table with the individual components has been added to the supplementary. Restricting the analysis to CCS patients alone only did minor changes to the point estimate (line 200).

| Indication | Non-CTO | Success | Unsuccess |
| --- | --- | --- | --- |
| Arrhythmia | 298 (3.3%) | 35 (2.7%) | 25 (9.2%) |
| PCI before valve surgery | 88 (1%) | 5 (0.4%) | 3 (1.1%) |
| Complication to CAG, PCI or CABG | 23 (0.3%) | 0 (0.05) | 0 (0.0%) |
| Non-anginal chest pain | 26 (0.3%) | 3 (2.3%) | 3 (1.1%) |
| PCI before non-cardiac surgery | 17 (0.2%) | 1 (0.1%) | 1 (0.4%) |
| Hybrid procedure after CABG | 91 (1.0%) | 3 (0.2%) | 3 (1.1%) |
| Missing | 108 (1.2%) | 15 (1.2%) | 7 (2.6%) |

**10. We noticed that a number of patients underwent non-CTO PCI, is in the same procedure or staged procedure, what’s the long-term impact of these non-CTO PCIs in CTO patients, What’s the rate of MI and revascularization could be attributed to non-CTO lesion?**

This is an excellent question. Patients with non-CTO TVR in the CTO groups was 19 (successful: 16 (1.2%) and unsuccessful: 3 (1.1%)). However, these were not included in the secondary endpoint of TVR presented in table 3 as this was only revascularizations that could be attributed to the target CTO. None (0%) of the MI in MACCE or the individual secondary end point was attributed to the non-CTO lesions in CTO patients. In the “any revascularization” which was included in MACCE 11/197 (5.6%) events were attributed to non-CTO TVR in the successful group and 3/32 (9.4%) in the unsuccessful group. Since there are only few events in the unsuccessful group it would not be meaningful to make independent time-to-event statistics for non-CTO TVR. However, we have made the following changes to the manuscript to improve transparency.

Added line 119: “TVR of non-CTO vessels treated at baseline were not adjudicated as TVR but as any repeat revascularization and were therefore also included in MACCE.”

Added line 177: “Nineteen CTO patients (successful: 16 (1.2%) and unsuccessful: 3 (1.2%)) had a TVR of a non-CTO vessel treated at baseline, and none of these lesions attributed to the secondary end point of MI.”
